# Filamentous Bacteriophage Fd as an Antigen Delivery System in Vaccination

**DOI:** 10.3390/ijms13045179

**Published:** 2012-04-24

**Authors:** Antonella Prisco, Piergiuseppe De Berardinis

**Affiliations:** 1 Institute of Genetics and Biophysics, CNR, via P. Castellino 111, 80131, Naples, Italy; 2 Institute of Protein Biochemistry, CNR, via P. Castellino 111, 80131, Naples, Italy

**Keywords:** vaccine, antigen delivery system, bacteriophage fd

## Abstract

Peptides displayed on the surface of filamentous bacteriophage fd are able to induce humoral as well as cell-mediated immune responses, which makes phage particles an attractive antigen delivery system to design new vaccines. The immune response induced by phage-displayed peptides can be enhanced by targeting phage particles to the professional antigen presenting cells, utilizing a single-chain antibody fragment that binds dendritic cell receptor DEC-205. Here, we review recent advances in the use of filamentous phage fd as a platform for peptide vaccines, with a special focus on the use of phage fd as an antigen delivery platform for peptide vaccines in Alzheimer’s Disease and cancer.

## 1. Introduction

A crucial challenge for vaccine development is to design vaccines that induce long-lasting protective immune responses without compromising safety and tolerability. In various settings, it is important to focus the immune response to defined B and T epitopes, which in some instances consist of short peptides that are not, in themselves, immunogenic. The induction of an effective immune response requires antigen uptake and processing by antigen-presenting cells (APC), T cell priming by activated APC, and activation of B and T cells. The antigen delivery system can confer immunogenicity to short peptides that are not by themselves immunogenic, and can overcome the limitations inherent to synthetic peptides in terms of stability and toxicity. The immunological properties of antigen delivery systems are a complex function of their size, geometry, kinetics and molecular patterns [1].

Filamentous bacteriophages are non-pathogenic, non-lytic viruses that are able to infect and replicate only in *Escherichia coli* cells carrying an F′ episome. Peptides can be chemically conjugated to the phage, or displayed as recombinant fusions to the coat proteins [2]. The immunogenicity of short peptide epitopes is enhanced when they are displayed on the phage capsid, chemical stability is increased [3], cloning and purification protocols required to produce the immunogen are easy, and costs are very low [4]. Phage-based products have been recently approved in food safety by the US Food and Drug Administration (FDA) [5]. Moreover, a phase 1 clinical trial approved by FDA in 2008 established the safety of a phage preparation consisting of a cocktail of phages to target bacteria in a venous leg ulcer, and cleared the way for more phage therapy trials [5,6].

Different kinds of bacteriophages (filamentous phage [7], lambda phage [8], T4 [9] and T7 [10]) can be utilized in phage-display vaccination and DNA vaccination (reviewed in [11]). In this review, we discuss recent advances in the development of filamentous bacteriophage fd as an antigen delivery system for B and T cell epitopes. Bacteriophage fd shares a 98% identity with the genomes of filamentous phages M13 and f1.

## 2. The Fd Bacteriophage

**Figure 1:**
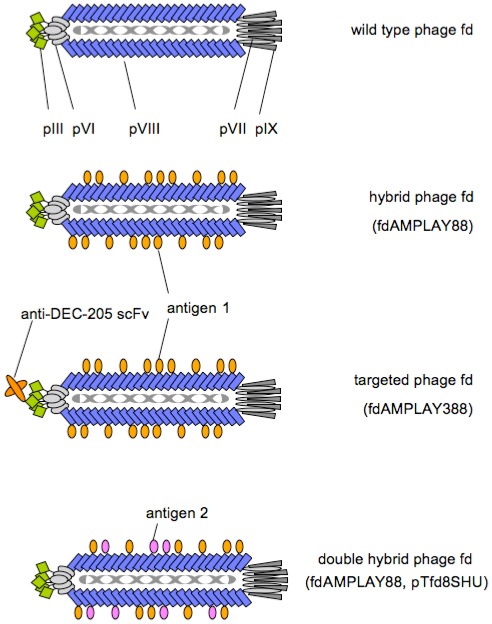
The structure of wild type page fd, and engineered phages. Hybrid phages based on vector fdAMPLAY88 display antigen on recombinant pVIII proteins, interspersed with wild type pVIII. fdAMPLAY388 vector allows the display of anti-DEC-205 on pIII protein. Double hybrid phages generated with fdAMPLAY88 and pTfd8SHU display two different antigens on the same virion.

The fd bacteriophage consists of a single-stranded DNA genome of about 6400 nucleotides surrounded by 2750 copies of a 50 residue α-helical protein, Major Coat Protein pVIII, which form a filamentous capsid, plus a few copies of minor proteins at the filament ends (Figure 1). At one end of the phage capsid there are five copies of the surface exposed pIII and its accessory protein, pVI, the first proteins to interact with the *E. coli* host during infection. The coat’s dimensions are flexible and the number of pVIII copies adjusts to accommodate the size of the single stranded genome it packages [12–15].

Since the first description of the phage display technique 25 years ago [16], filamentous bacteriophages have been largely employed for the generation of peptide libraries, based on phage virions displaying peptides encoded by degenerate oligonucleotide sequences that have been cloned into a gene coding for one of the viral coat proteins [12–14]. Thanks to the development of modified phage genomes, directional cloning of the sequence of interest as a fusion to protein pVIII or pIII is now very easy: synthetic complementary oligonucleotides encoding the sequence of interest can be readily annealed and ligated into the phage genome, cut with restriction enzymes.

Most of the data described here regards phages generated by utilizing the fdAMPLAY88 vector [17], a phage genome modified to include an origin of replication in *Escherichia coli*, the β-lactamase gene encoding resistance to antibiotic ampicillin, and an additional copy of the gene encoding pVIII, including restriction sites that allow easy directional cloning of exogenous sequences as fusions at the *N*-terminus of pVIII. As bacterial colonies transformed with fdAMPLAY88 can be selected by antibiotic resistance, peptide-displaying phages can be generated by utilizing the very same basic microbiology techniques utilized for any plasmid vector [18].

The procedure to purify filamentous bacteriophages, to utilize them as immunogens, is very simple [14]. Phages can be precipitated from the supernatant of infected bacterial cultures with 20% PEG6000 and 2.5M NaCl, and then can be further purified from bacterial debris by a cesium chloride gradient. LPS, a component of the wall of gram negative bacteria that can heavily contaminate crude phage preparations, can be removed by multiple extractions with detergent Triton-X114 [19].

### 2.1. Antigen Delivery via Filamentous Bacteriophage Fd

Virtually all the phage proteins can be used to express exogenous amino acid sequences on the coat surface, fused to the *N*-terminal portion of each protein. The two proteins most often used are the pIII and the pVIII. pIII is about 400 amino acids long and is involved in phage-host interaction during infection. pVIII is 50 amino acids long, and is the most abundant phage protein, making up the long filamentous capsid of the phage. Recombinant virions, carrying multiple copies of foreign peptides as fusion on all copies of pVIII, can be generated by cloning a DNA fragment encoding the peptide at the 5′ terminus of gene VIII in the double strand form of the phage genome. The use of pVIII as a peptide display is limited by the size of foreign peptides that can be displayed on every copy of the pVIII coat protein. Peptides longer than 6 amino acids may interfere (depending on the peptide) with the coat protein functions in viral packaging and bacterial infectivity [18,20]. To overcome this problem, the production of hybrid virions is possible, in which the exogenous peptide is displayed only on a fraction of the copies of pVIII [20]. With this approach, the size of the peptide displayed on pVIII can be increased up to 14–20 amino acids. In this hybrid phage display system, the viral gene encoding for the fusion coat protein can be carried on a phagemid, a plasmid that contains the phage origin of replication and the phage packaging signal. The wild type coat proteins and all the proteins for the phage assembly are provided by a helper phage. Infection of a bacterial host containing a phagemid with a helper phage provides the necessary viral components to enable single stranded DNA replication and packaging of the phagemid DNA into phage particles. A helper phage is used that lacks the phage packaging signal (for example a M13 derivative), so that it packages less efficiently than the phagemid, and the phage particles that are generated will predominantly contain phagemid DNA [20].

Alternatively, the need for an helper phage and superinfection can be obviated by using a modified phage vector such as vector fdAMPLAY88 carrying two copies of gene VIII: a wild type gene VIII, and a modified gene VIII containing two unique restriction sites at the 5′ terminus [17]. This vector allows the production of hybrid phages by enabling the incorporation of the chimeric pVIII proteins interspersed with wild type coat proteins during the assembly of phage particles (Figure 1).

A further modification of this technique has allowed the simultaneous display of two different peptides on the surface of the same hybrid phage particles, using *E. coli* cells transformed with a plasmid conferring tetracycline resistance and providing another modified gene VIII (plasmid pTfd8SHU, [17]). Cells transformed with this plasmid, when infected with the bacteriophage fdAMPLAY88 produce virions simultaneously displaying two different peptides (Figure 1).

The major limitation to the use of phage display on pVIII protein as an antigen delivery system in vaccination is the fact that some peptides are displayed at a low copy number (less than 5% of total pVIII), due to inefficient incorporation of the recombinant protein in the phage capside. Moreover, incorporation of recombinant pVIII proteins within the phage capside does not guarantee a strong humoral response to the displayed antigen. As an example, phage fdAD(23–29), displaying peptide DVGSNKG, only induced an antibody response to peptide DVGSNKG in one out of 12 immunized mice, despite the fact that the recombinant pVIII protein accounted for 25% of total pVIII in the phage capside [21].

Importantly, NMR spectroscopy studies have shown that peptide GPGRAF, the principal neutralizing determinant of HIV-1, when inserted near the N terminus of the pVIII protein of bacteriophage fd adopts a double bend S-shaped conformation similar to the antibody-bound structure determined by X-ray crystallography [22]. As solution NMR studies of the GPGRAF sequence embedded in 12 to 40 residue polypeptides failed to identify a persistent three-dimensional conformation [22], as is typically the case for short peptides in aqueous solution, this observation implies that a short peptide, when displayed on the bacteriophage coat protein, can in some instances have an enhanced propensity to adopt a conformation similar to that found in the native protein from which it is derived [22].

### 2.2. The Effect of Phage Display on Antigen Uptake and Processing

Soluble antigens efficiently enter lymph vessels, but are inefficiently up taken by antigen presenting cells (APC), whereas particulate antigen is up taken more efficiently [1]. Bacteriophage fd capsids are cylindrical flexible protein scaffolds, approximately 7 nm wide, and 890 nm long, and are efficiently taken up and processed by antigen presenting cells [23].

The fate of processed bacteriophage proteins has been followed using confocal microscopy in human B cell lines exposed to fluorescently labeled phage particles. Antigens endocytosed by APCs undergo proteolysis in the endosomal–lysosomal compartments and are loaded on MHC class II molecules, but can also be loaded on MHC class I, in the endoplasmic reticulum, by a process known as cross-presentation. Phage-displayed peptides are able to activate antigen-specific CD4^+^ T cells [24]. Peptides derived from the bacteriophages reach both the major histocompatibility complex (MHC) class II compartment and the endoplasmic reticulum, and can be loaded both on MHC class I and class II [23]. Since MHC class II molecules stimulate CD4^+^ T cells, and MHC class I molecules stimulate CD8 T cells, this remarkable feature may explain the ability of bacteriophages displaying foreign T-cell epitopes to prime strong T-helper-dependent cytotoxic T cell responses [25,26], an important feature for vaccines against viral infections and cancer. The particulate nature of phage-displayed antigens, and the size of phages, that falls within the size range that optimizes cross presentation, most probably underlie the remarkable effects of phage-display on antigen uptake and processing [1,27].

### 2.3. The Immune Response to Phage Fd

Vaccination of mice with filamentous phage fd induces a robust anti-phage antibody response, after a single immunization, even without adjuvant [2,21,28]. Antisera are able to recognize intact phage particles in phage ELISA.

The epitopes recognized by three mouse monoclonal antibodies, raised against whole filamentous phage fd particles [29], have been mapped to the first 12 residues of pVIII. A polyclonal rabbit antiserum was also shown to bind to this *N*-terminal region [30]. In particular, the epitope of antibody B62-FE2 has been mapped to the first 9 amino acid residues of PVIII, with an essential contribution to binding by residues Ala1, Asp4, Asp5 and Lys9 [30]. In electron microscopy experiments, this antibody appears uniformly bound along the whole axis of the phage particles, suggesting that the epitope of pVIII that it recognizes is accessible on the surface of the phage capside [30].

## 3. Cytotoxic T Cell (CTL) Responses to Peptides Exposed on Bacteriophages

It is assumed that a CTL response is necessary for effective clearance of virus infected cells and is implicated in the control of the expansion of tumor cells. In this context, we have shown that fd virions displaying peptide RT2 (ILKEPVHGV), corresponding to residues 309–317 of the reverse transcriptase (RTase) of HIV-1, are able to prime a CTL response specific for this HIV-1 epitope in human cell lines [25]. HLA-A2 transgenic mice immunized with bacteriophage virions displaying peptide RT2 mount an effective, specific anti-HIV-RT2 CTL response [25].

Engineered bacteriophages fd very effectively elicit specific CTL primary responses to tumor associated antigens (TAAs), as demonstrated by our studies on two HLA-A2 restricted CTL epitopes, MAGE-A3_271–279_ and MAGE-A10_254–262_, from tumor associated antigens MAGE-A3 and MAGE-A10. Early studies on T-cell responses to MAGE-A3_271–279_ and MAGE-A10_254–262_ peptides, even in association with cytokines or presented by dendritic cells as antigen-presenting cells (APCs), have shown that specific CTL responses required repeated stimulations *in vitro* [31–33], and that repeated immunizations rarely generated CTL responses *in vivo* [34–36]. Moreover, when the generation of peptide-specific CTLs could be achieved, CTLs might fail to recognize the peptide epitopes on the neoplastic cell such as in the case of one of the MAGE epitope (MAGE-A3_271–279)_ which we displayed on the phage particles [37]. These observations have raised concerns on the immunogenicity of these MAGE peptide epitopes and, hence, on their usefulness as vaccines. Therefore, the possibility of delivering TAA peptides in a highly immunogenic form, capable of eliciting not only specific CTLs but also CTL responses potent enough to recognize low amounts of antigen on the tumor cell, represents one of the key issues for the development of effective peptide-based cancer vaccines. We reported that engineered filamentous bacteriophages fd, displaying tumor antigens, are able to induce strong anti-tumor CTL responses, both *in vitro* and *in vivo* [26]. In particular, we constructed two double hybrid bacteriophage virions, fd23/Mg10 and fd23/Mg3, co-expressing promiscuous HLA-DR-restricted helper T-cell peptide pep23 and either the HLA-A2 restricted CTL peptide MAGE-A10_254–262_ or MAGE-A3_271–279_ on the same capsid. We showed that a single stimulation of PBMCs from HLA-A2^+^ healthy donors with autologous antigen presenting cells pulsed with fd23/Mg10 or fd23/Mg3 virions elicited strong peptide-specific CTL responses *in vitro*. Moreover, a single immunization of HHD (HLA-A2^+^/H-2D^b+^) transgenic mice with fd23/Mg10 or fd23/Mg3 virions generated peptide-specific CTLs in splenocytes restimulated *in vitro* once with syngeneic peptide-loaded antigen presenting cells. Finally, we have shown that fd23/Mg3 virions inhibit the growth of tumor cells expressing the MAGE A3 or A10 antigens *in vivo* in immunized mice [26]. Mice were immunized with two doses of fd23/Mg3 hybrid phages at a 3 weeks interval. Seven days after the second immunization, mice were challenged with EL-4-HHD/MAGE-A3 tumor cells, and the incidence and growth of tumor, as well as animal survival, were monitored. A significant protection was observed in mice vaccinated with fd23/Mg3 virions compared with the controls, with up to 40% tumor-free animals 80 days after challenge [26]. Overall, these results indicate that engineered bacteriophages fd represent a TAA peptide delivery system that very effectively elicits specific CTL primary responses [31–37].

## 4. Targeting Phage Particles to Dendritic Cells

The efficacy of filamentous bacteriophage fd antigen delivery system can be further improved by targeting the phage particles to the “professional antigen presenting cells”. In particular, we engineered the minor pIII bacteriophage coat protein in order to express at its *N*-terminus an antibody fragment able to specifically target phage virions to a receptor expressed on dendritic cells. It is known that pIII proteins allow the display of long peptides, including antibodies [38]. Phage libraries displaying single chain antibody fragments (scFv) at the *N*-terminus of the minor pIII coat proteins can be produced with a diversity > of 6.5 × 10^6^ [39]. Thus, phage particles were genetically engineered to express on their coat, as fusion with the pIII protein, an scFv that recognizes the DEC-205 receptor present on the membrane of dendritic cells. To display a foreign sequence at the *N*-terminus of the fd bacteriophage pIII protein, we have modified the 5′-terminus of the pIII gene of the previously described fdAMPLAY88 vector [18] by site-directed mutagenesis, in order to introduce two unique restriction sites, XhoI and SpeI, which do not change the amino acid sequence of the expressed pIII protein. In this way we generated the new fdAMPLAY388 vector, which is suitable for the cloning and expression of foreign sequence between the +4 and +5 amino acidic residues of the mature pIII protein. We thus inserted the sequence coding for the single chain variable fragment (scFv) of the NLDC-145 mAb, which is known to bind the mouse dendritic cell restricted surface molecule DEC-205 [40,41]. The single-chain variable fragment is constituted by the variable regions of heavy and light chains of the anti-DEC-205 antibody assembled with a (GGGGS)_3_ encoding linker. The DNA coding for the anti-DEC-205 scFv was cloned into the gpIII of the fdAMPLAY388 filamentous phage to produce fdsc-aDEC phage particles which are able to bind the mouse dendritic cell surface molecule DEC-205 (Figure 1). It is known that dendritic cells play a central role in the induction of antigen specific immune responses and that presentation of vaccine candidate molecules by DC may be enhanced by targeting antigenic determinants via DC receptors [42]. We demonstrated that DC-targeting with fd particles, double-displaying the anti-DEC-205 fragment on the pIII protein and the OVA_257–264_ antigenic determinant on the pVIII protein, induced potent inhibition of the growth of the B16-OVA tumor *in vivo*. Immunization with DEC-205-targeted phages induced stronger response than other immmunization strategies, being comparable to the response induced by adoptively transferred DCs. Since targeting DEC-205 in the absence of DC activation/maturation agents had previously been described to result in tolerance, the ability of fd bacteriophages to induce a strong tumor specific immune response by targeting DCs through DEC-205 further validate the potential employment of this safe, versatile and inexpensive delivery system for vaccine formulation.

Finally, we also found that fd particles displaying specific CTL epitopes can be used to perform delayed type hypersensitivity (DTH) reaction, and this may allow the *in vivo* monitoring of immune responses mediated by antigen-specific CD8^+^ T cells [43]. In a previously reported study [44], fd virions were used to sensitize mice and then to induce DTH reaction. We used fd virions only to challenge the DTH in mice sensitized by the administration of a combination of synthetic helper and CTL peptides. In this way we proved that fd bacteriophages are able to stimulate in the tissue the specific CD8^+^ T lymphocytes which were primed by a different delivery of the antigenic determinant. DTH reactions in the skin have been observed in many immunotherapy protocols and are often used as an indicator of anti-tumor immunity and virus vaccine efficacy [45]. In addition, since DTH is relatively straightforward to perform, it may be employed as a preliminary screen for diagnostic virus infection, and may serve as an *in vivo* measure of the lymphocytes trafficking to sites of infection [24,46].

## 5. Antibody Responses to Pathogen Epitopes Exposed on Bacteriophage Fd

Several reports have described the use of filamentous bacteriophage as immunogen carriers for raising antibodies against peptides and proteins displayed on their surface. Bacteriophages displaying a disease-specific protective epitope can be utilized as a vaccine to confer protection against infection.

Two peptide epitopes of the circumsporozoite protein of *Plasmodium falciparum*, the parasite responsible for malaria, have been displayed on filamentous bacteriophages, namely epitope MAL1, consisting of sequence NANPNANPNANP, and epitope MAL2, consisting of sequence NDDSYIPSAEKI. In various strains of mice, phage-displayed malaria epitopes induce a specific IgG antibody response, with no need for adjuvant. Experiments in nude (nu/nu) and heterozygote (nu +/−) BALB/c mice have demonstrated that the immune response is T-cell dependent. Interestingly, peptide MAL1 adopts a single, stable conformation when displayed on the phage surface [47,48]. Peptide sequences from the V3 loop of the surface glycoprotein gp120 of HIV displayed on phage fd are recognized by human HIV antisera. Immunization induces high titres of antibodies against the V3 loop in mice, which have virus-neutralizing properties [49]. The amino acid sequence of HIV reverse transcriptase (RT) from residue 248 to residue 262, expressed on the surface of filamentous phage fd, was recognized by the T-cells and induced production of Abs [24]. Immunization of mice with a bacteriophage displaying, on the pIII protein, protective epitope 173–187 from the glycoprotein G of the human Respiratory Syncytial Virus (RSV) induces a high level of circulating RSV-specific antibodies, and confers resistance to RSV infection [50].

## 6. Antibody Responses to β-amyloid Epitopes Exposed on Bacteriophage Fd

Alzheimer’s Disease (AD) is a neurodegenerative disease, causing memory loss and dementia, characterized by the deposition, in the brain, of insoluble proteic aggregates, the amyloid plaques, that are mainly composed of β-amyloid peptide [51]. Active and passive immunization studies performed in transgenic mouse models of β-amyloid deposition have demonstrated that antibodies against β-amyloid are able to reduce amyloid load and improve cognition [52,53]. The development of an effective and safe immunotherapy protocol for Alzheimer’s Disease faces two big challenges, namely overcoming the low immunogenicity of the β-amyloid peptide and avoiding detrimental autoimmune responses. T-cell mediated adverse reactions were observed in humans immunized with the whole β-amyloid peptide [54]; while the antigen specificity of the lymphocytes that caused the adverse response are unknown, immunogens that are devoid of β-amyloid T epitopes are considered in principle safer.

Filamentous bacteriophages are an effective antigen delivery system to induce antibody responses to short fragments of β-amyloid, that do not include T cell epitopes.

The table reports the beta-amyloide epitopes that have been displayed on filamentous phage fd, the site of insertion of each epitope within pVIII, and the number of copies of epitope per phage particle. Small variation in the sequence of the epitope and in the insertion site can bring significant changes in the epitope copy-number. The wild type *N*-terminal amino acid sequence of the pVIII protein encoded by fdAMPLAY88 is also reported.

A phage-based anti-β-amyloid vaccine was generated by the group of Beka Solomon, and consists of a phage displaying the EFRH epitope of β-amyloid. Several phages displaying the EFRH epitope were selected from a random 15-mer peptide phage library for their ability to bind anti-β-amyloid antibody 6C6, a mouse monoclonal that has the ability to disaggregate β-amyloid fibrils *in vitro* [55]. The selected phages had the ability to elicit antibodies with the same disaggregating properties as 6C6 upon immunization. Antibody titers, however, were quite low, possibly because part of the antibody response was directed at phage proteins and at irrelevant epitopes within the displayed 15-mer peptide. Solomon and collaborators improved the system by generating phages that only display the EFRH epitope. Phages expressing 300 copies of the peptide, obtained by insertion of a tandem repeat of the EFRH peptide, were more immunogenic than phages expressing 150 copies of the peptide, suggesting that epitope density was a limiting factor within this immunization protocol [56]. EFRH phages however elicit an immune response against β-amyloid that leads to a reduction of the amyloid load and improves cognition [55–59].

We further pursued B. Solomon’s strategy of using filamentous phages as a carrier for anti-amyloid immunization. In particular, we set out to compare the immunogenicity of different regions of β-amyloid in a phage carrier system, in order to determine which region of β-amyloid would be best suited for inclusion in a phage-based anti-amyloid vaccine. As the number of epitopes displayed per phage particles can influence the magnitude of the immune response, the inability to display some peptides at high levels is a limiting factor in the development of phage-based vaccines. We hypothesized that a simple way to obtain highly immunogenic “Amyloid Display” phages would be to promote a high epitope density by decreasing epitope size, and we minimized the number of exogenous amino acids on phage protein pVIII by taking advantage of short identities (1 or 2 amino acids) between the *N*-terminus of phage protein pVIII and the epitopes of interest. In the case of phage fdAD(1–7), sequence DAEFRH was inserted between amino acid residue 3 and 4 of pVIII. The 4th amino acid of pVIII being a D, this resulted in sequence DAEFRHD (Table 1). In the case of phage fdAD(2–6), we took advantage of the natural *N*-terminal sequence of the mature pVIII protein of the phage to recreate 5aa epitope “AEFRH” by inserting only 3 exogenous amino acids after the endogenous AE sequence of pVIII (Table 1). We have thus obtained phages that display 300 to 800 copies of amyloid epitope per phage particle, depending on the specific epitope. In particular, epitope 2–6 (AEFRH), that is nearly identical to the EFRH epitope analyzed by Solomon at a maximum expression level of 300 copied per phage particle [56], was expressed at 810 copies per phage particle (Table 1). We also included in our analysis sequence 4–11 of β-amyloid, that is known to be the target of therapeutically effective antibodies against β-amyloid that inhibit cytotoxicity and fibrillogenesis [60], and sequence 23–29, that was identified in structural studies as a turn between the β-strands typical of the aggregated β-amyloid structure ([61] and references therein) (Table 1).

We obtained antisera to all the phage-displayed β-amyloid epitopes. Responder antisera significantly cross-reacted with full-length, pre-aggregated β-amyloid. The most represented isotype was IgG2b (over 50%), with IGg2a, and IgG3 also being present in significant amounts (around 20%). The high (10-fold) ratio of IgG2a to IgG1 suggests that a TH1-like T cell response was induced. Indeed, fdAD(2–6) induces significant secretion of IFN-γ in lymphocyte cultures from mice immunized with fdAD(2–6) [19]. Nevertheless, we observed a marked difference in the immunogenicity of the β-amyloid epitopes displayed by the 4 fdAD phages. Most of the mice that received fdAD(4–11) or fdAD(23–29) did not produce antibodies against the displayed β-amyloid epitope, and the few responders had low titers. Instead, two injections of phages fdAD(1–7) or fdAD(2–6) were sufficient to obtain anti-β-amyloid antibodies in 100% of immunized mice, representing a significant improvement over the performance of previously reported anti-β-amyloid immunization protocols, including phage-based immunizations, where several injections of antigen were necessary to observe a response, and only a fraction of immunized individuals ever produced anti-β-amyloid antibodies [58]. As little as 1.5 μg of phage-carried peptide was sufficient to obtain a maximal antibody response after two injections of antigen, and the circulating anti-β-amyloid antibodies persisted for at least 8 months after antigen injections were interrupted.

Antibody titers elicited by phage fdAD(2–6) double those elicited by fdAD(1–7) when mice were injected with equal amounts of phage antigen. Dose response curves revealed that the difference could not be attributed to the small difference in the molar amount of exogenous peptide carried by the two phages. We conclude that peptide 2–6, in the context of phage fdAD(2–6), is more immunogenic than peptide 1–7, in the context of phage fdAD(1–7). Since epitope 2–6 is fully contained within epitope 1–7, our data demonstrate that natural flanking sequences can reduce the immunogenicity of a B cell epitope. As in other instances it has been shown that increasing the length of a B epitope inserted in a fusion protein increases its immunogenicity [62], experimental testing of the effects of flanking sequences is potentially useful to optimize immunogenicity.

We have observed a significant reduction in the number of β-amyloid plaques in the hippocampus and cortex of 8-month-old Alzheimer’s Disease model mice immunized monthly with fdAD(2–6) from age 2 months, suggesting that the treatment effectively delays the onset of plaque pathology [21]. The amount of brain β-amyloid is also reduced when aged sixteen-month-old Alzheimer’s Disease model mice are immunized with the EFRH-phage for a five month period [58]. Importantly, Alzheimer’s Disease model mice treated with phage-EFRH show a considerable improvement in their cognitive behavior in the Morris Water Maze test [56].

Overall, data from anti-β-amyloid immunization experiments show that phage-based antigens represent a good strategy to focus the immune response to a defined B cell epitope, and obtain antibody levels that afford a therapeutic effect. On the other hand, we observed marked differences in the immunogenicity of different β-amyloid epitopes displayed by the phages, despite incorporation of recombinant pVIII proteins in the phage capsides.

## 7. Conclusions

Because of their ability to display exogenous peptides on their surface as a fusion to phage proteins, bacteriophages fd may represent a powerful antigen delivery system, that can be utilized to develop safe and inexpensive vaccines. Phage based antigens are suitable for the induction of antibody responses and T cell responses. Bacteriophages are easy to prepare in large quantities and at low cost and are very stable, although crude phage preparations are heavily contaminated with lipopolysaccharide (LPS), making careful purification from LPS essential for use of phages as a vaccine.

One of the major limitations of genetically engineered phages as an antigen delivery system is the fact that some peptides cannot be displayed at high copy-number on the phage capside, which poses limits to the length and amino acid sequences of the epitopes that can be delivered. Nevertheless, the immunogenic properties of phages can be exploited also in the case of peptides that cannot be efficiently displayed on the surface of the phage as a fusion to phage proteins, as the surface of the phage is suitable for the chemical conjugation of peptides and proteins. The immune response to filamentous phage fd has been described to involve cytokine interferon-γ, thus the use of phage fd is of particular interest in immunization procedures in which this type of response is desirable.

The general applicability of a peptide vaccine aimed at eliciting a T cell response to a specific CD4 or CD8 T cell epitope is limited by the HLA diversity in the human population, as the presentation of the T cell epitope included in the vaccine can be restricted by a specific haplotype. Thus, a T cell vaccine, to be of general use, should include T cell epitopes that display promiscuous binding to HLA haplotypes [63]. In the case of phage-based vaccines, an alternative to the use of HLA-promiscuous epitopes would be the production of a mixture of different bacteriophages, each delivering a T cell epitope restricted by a different haplotype. In the case of antibody responses, a general limitation of vaccines based on short peptides is that only some B cell epitopes are linear epitopes. On the other hand, non-linear epitopes can be mimicked by “mimotope” peptides, that can reproduce the epitope despite little or no amino acid sequence homology with the antigen, and are able to induce an antibody response that cross-reacts with the original antigen. Mimotope peptides have been often identified by the screening of phage display libraries (reviewed in [64–66]). Overall, filamentous bacteriophage fd represents a promising antigen delivery platform for the development of peptide vaccines, deserving further research.

## Figures and Tables

**Table 1:** Beta-amyloid epitope location and copy number in phages utilized as immunogens.

Immunogen	Epitope	pVIII Sequence	Epitope Copy Number	Reference
C3-II	EFRH	VHEPH**EFRH**VALNPV	n.a.	[55]
BS-Y	EFRH	n.a.	150	[56]
BS-12	EFRHEFRH	n.a.	300	[56]
fdAD(1–7)	DAEFRHD	AEG**DAEFRHD**D	594	[21]
fdAD(2–6)	AEFRH	**AEFRH**GDD	810	[21]
fdAD(4–11)	FRHDSGY	AEG**FRHDSGYE**DD	297	[21]
fdAD(23–29)	DVGSNK	AEG**DVGSNKG**DD	675	[21]
fdAMPLAY88		AEGDD	0	[17]
